# Life weariness, suicidal thoughts and mortality: a sixteen-year longitudinal study among men and women older than 60 years

**DOI:** 10.1186/s12889-021-11329-z

**Published:** 2021-07-09

**Authors:** Cecilia Fagerström, Anna-Karin Welmer, Sölve Elmståhl, Hanna Tuvesson

**Affiliations:** 1 Department of Health and Caring Sciences, Faculty of Health and Life Sciences, Linnaeus University, Kalmar, Sweden; 2 The Research Section, Region Kalmar County, Kalmar, Sweden; 3 Department of Neurobiology, Care Sciences and Society, Aging Research Center, Karolinska Institutet and Stockholm University, Stockholm, Sweden; 4 Division of Physiotherapy, Department of Neurobiology, Care Sciences and Society, Karolinska Institutet, Stockholm, Sweden; 5 Division of Geriatric Medicine, Department of Clinical Sciences in Malmö, Lund University, Malmö, Sweden; 6 Department of Health and Caring Sciences, Faculty of Health and Life Sciences, Linnaeus University, Växjö, Sweden

**Keywords:** Life weariness, Longitudinal study, Old age, Suicidal thoughts

## Abstract

**Background:**

Suicide in old age is a significant contributor to mortality. However, the extent to which life weariness and suicidal thoughts impact on mortality in a long-term perspective is unknown. The aim of this study was to investigate the effect of life weariness and suicidal thoughts on long-term survival (16 years) in an older Swedish population, controlling for demographic and social network factors and depression. A further aim was to investigate differences in sex and age interactions in relation to mortality among individuals with and without life weariness and suicidal thoughts.

**Methods:**

A longitudinal cohort study on a national, representative sample of individuals aged 60+ years was conducted within the Swedish National Study of Aging and Care study. The sample included 7213 individuals, who provided information about life weariness and suicidal thoughts through an item derived from the Montgomery-Åsberg Depression Rating Scale. Data were analysed with multivariate Cox proportional hazards models, adjusted for potential confounders.

**Results:**

At baseline, 12.5% of the participants (14.6% of females and 9.5% of males) reported life weariness and suicidal thoughts. During the 16-year follow-up, a mean survival time was 11.5 years (standard deviation (SD) 5.6), and 3804 individuals died (59.5% females and 40.5% males). Individuals with life weariness and suicidal thoughts had half the survival rate compared with those without such thoughts (24.5% vs. 50.6%), with a mean survival time of 8.4 years (SD 5.7) versus 12.0 years (SD 5.4). The multi-adjusted hazard ratio of mortality for those reporting life weariness and suicidal thoughts was 1.44 (95% confidence interval, 1.30–1.59), with the population attributable risk at 11.1%. In the models, being male or female 80+ years showed the highest multi-adjusted hazard ratio of long-term mortality (ref. female 60–69 years).

**Conclusions:**

The findings suggested that life weariness and suicidal thoughts were risk factors for long-term mortality, when controlled for sex and age interactions that were found to strongly predict long-term mortality. These findings have practical implications in prevention of mortality, emphasising the importance of screening, identifying, and intercepting older men and women with signs of life weariness and suicidal thoughts.

**Trial registration:**

Not applicable.

## Background

Suicide rates in late life are high in most countries, making suicide a major public health concern worldwide [1–3]. Globally, the all-age suicide rate is 11.1 deaths per 100,000 [4]. In Sweden, the rate is even higher (15 deaths per 100,000), and differs greatly between the sexes in the age group 65–84 years (24.4 deaths per 100,000 in men and 7.3 per 100,000 in women) [5]. Early signs of suicidality, such as suicidal thoughts, have been suggested as being among the most prevalent predictors of completed suicide [6, 7], but little is known regarding the long-term effect on mortality of suicidal thoughts in late life.

The spectrum of suicidality ranges from life weariness and suicidal thoughts to suicide attempts and completed suicide [8, 9]. Several concepts are used in the literature to describe initial or early signs of suicidality, such as suicidal feelings, suicidal ideation, and suicidal thoughts (see for example [10–12]). In this study, we used the term “life weariness and suicidal thoughts”, comprising suicidal aspects such as life weariness, death wishes, suicide preparation, and suicide planning [13].

To our knowledge, there are few long-term longitudinal studies that have investigated the impact of life weariness and suicidal thoughts on mortality within national, representative samples of older adults. One 5-year mortality study from 2010 found that the “wish to die” was associated with mortality in usual care of older adults who were primary health care patients (*n* = 1202) [14]. Another study from 2013 presented data from 861 community-based older adults (70+), followed during 17 years, and found that suicidal ideation increased the risk of death from natural causes by 23% [15]. For several reasons, it is important to emphasise the potential impact of life weariness and suicidal thoughts on mortality. Older adults with life weariness and suicidal thoughts could risk a decline in both physical and mental health through, for example, reduction of disease management, treatment adherence, health care visits, physical activities, or food intake. It is reasonable to assume that detection of life weariness and suicidal thoughts, if it results in appropriate actions, referrals, and follow-ups, could help prevent mortality in older adults.

In this study, we considered several factors that might be relevant in explaining the risk of death in older adults with suicidal thoughts: demographic factors, social network factors, and depression. Much of the previous research regarding these factors in older adults is cross-sectional and provides a limitative snapshot of life weariness and suicidal thoughts in late life. Regarding demographic and social network factors, longitudinal research suggests that age, sex, marital status, education, financial situation, living situation, and country of origin are significantly associated with suicidal thoughts (see for example [6, 7, 10, 16, 17]). Depression has also been found to be associated with suicidal thoughts in older adults (see for example [11, 18]). A comprehensive model in which several of these variables are simultaneously accounted for can reveal whether specific variables exacerbate vulnerability among older adults with life weariness and suicidal thoughts. Furthermore, focusing on sex and age and accounting for their interaction could provide information that would be important to consider when developing preventive interventions targeting the older population.

The current study was intended to address a gap in the literature: the limited research in national, representative samples of older adults with long-term longitudinal data on the impact of life weariness and suicidal thoughts on mortality. Thus, the aim of the study was to investigate the effect of life weariness and suicidal thoughts on long-term survival (16 years) in an older Swedish population (60+), controlling for demographic and social network factors and depression. A further aim was to investigate differences in sex and age interactions in individuals with and without life weariness and suicidal thoughts.

## Methods

### Sample

Individuals included in this longitudinal study participated in the Swedish National Study on Aging and Care (SNAC), which was initiated in 2001 at centres in several parts of Sweden. The SNAC is a prospective multicentre study and includes randomly selected individuals in 10 age clusters (age in years: 60, 66, 72, 78, 81, 84, 87, 90, 93, 96+), drawn from the national population register. All individuals in the oldest age groups (i.e., 81–96+) were invited, with an oversampling process used due to the decreasing numbers of potential participants. More information about the SNAC study can be read elsewhere [19].

At baseline, during the period 2001–2004, participants (*n* = 7418) were recruited from geographical areas covering a medium-sized town, small towns, rural areas (five municipalities in the Skåne region and one municipality in Blekinge, all located in southern Sweden), and one urban district in Sweden’s capital, Stockholm. The participants were invited to the project by post and reasons for non-participation were registered. For the present study, those participants who gave information about life weariness/suicidal thoughts at baseline were included (*n* = 7213) and followed for up to 16 years. The follow-up period began at the time of each individual’s baseline examination and ended after 16.4 years (i.e., 6000 days) or at time of death.

The study was conducted in accordance with the ethical principles of the Helsinki Declaration and both verbal consent and written informed consent were obtained. The SNAC study was approved by the Ethics review boards of Lund University, LU 128 00, LU 650–00, LU 744–00 and Karolinska Institutet, Dnr 01–114.

### Data collection

The data collection team included trained nurses, physicians, and test leaders. Participants who could not come to the research centres were offered home visits in order to avoid selection bias. Data on participant age, sex, education, living arrangements, marital status, geographical residence, financial situation (two items), country of birth, self-reported depression (one item), social support (three items), and religious beliefs were obtained through single-item self-administered questionnaires (see Table 1 for details regarding items and response options).

**Table 1:** Descriptive characteristics of population at baseline (*n* = 7213) and 16-year follow-up, by mortality group

Variables	Baseline (***n*** = 7213)	Survivors (***n*** = 3409)	Deceased (***n*** = 3804)	***p*** value
**Age group** (%)				
60–69 years	3147 (43.6)	2540 (74.5)	607 (16.0)	< 0.001^3^
70–79 years	1754 (24.3)	709 (20.8)	1045 (27.5)	
> 80 years	2312 (32.1)	160 (4.7)	2152 (56.5)	
Mean age (SD)	73.11 (10.72)	65.74 (6.75)	79.72 (9.21)	
**Sex**				
Female (%)	4291 (59.5)	2029 (59.8)	2262 (52.7)	< 0.001^1^
**Sex-age interaction (%)**				
Male 60–69 ys	1474 (20.4)	1128 (33.2)	346 (9.1)	< 0.001^1^
Male 70–79 ys	707 (9.8)	213 (6.2)	494 (13.0)	
Male 80+ ys	741 (10.3)	39 (1.1)	702 (18.5)	
Female 60–69 ys	1673 (23.2)	1412 (41.5)	261 (6.9)	
Female 70–79 ys	1047 (14.5)	496 (14.5)	551 (14.5)	
Female 80+ ys	1571 (21.8)	121 (3.5)	1450 (38.0)	
**Marital status (%)**				
Married	3520 (49.9)	2117 (62.5)	1403 (38.1)	< 0.001^1^
Widow/-er	1890 (26.8)	385 (11.4)	1505 (40.8)	
Unmarried	708 (10.0)	320 (9.5)	388 (10.5)	
Divorced	788 (11.1)	458 (13.5)	330 (9.0)	
Living apart	169 (2.2)	105 (3.1)	60 (1.6)	
**Country of birth (%)**				
Sweden	6503 (91.3)	3045 (89.5)	3458 (92.9)	0.001^1^
Other Nordic country	222 (3.1)	112 (3.3)	110 (3.0)	
Other European country	335 (4.7)	211 (6.2)	124 (3.3)	
Outside Europe	62 (0.9)	33 (1.0)	29 (0.8)	
**Geographical area (%)**				
Urban	4958 (68.8)	2569 (75.3)	2389 (62.8)	< 0.001^1^
Medium-sized town	2158 (29.9)	800 (23.5)	1358 (35.7)	
Rural	97 (1.3)	40 (1.2)	57 (1.5)	
**Highest level of education (%)**				
Primary school, left at age < 13 yrs	2268 (32.7)	763 (23.0)	1505 (41.5)	< 0.001^2^
Secondary school, left at age 14–16 yrs	831 (12.0)	379 (11.4)	452 (12.5)	
Upper secondary or vocational school, left at age 18–19 yrs	2252 (32.4)	1123 (33.9)	1129 (31.2)	
College or above	1589 (22.9)	1053 (31.7)	536 (14.8)	
**Housing (%)**				
Community dwelling	6816 (96.2)	3362 (99.2)	3454 (93.5)	< 0.001^1^
Residential care facility and other	267 (3.8)	26 (0.8)	241 (6.5)	
**Financial difficulties in the last year**				
Yes	373 (5.3)	1173 (5.1)	200 (5.5)	0.453^1^
**Able to access EUR 1,400 within a week (%)**				
Yes	6043 (87.6)	3042 (90.6)	3001 (84.8)	< 0.001^1^
**Depression (%)**				
Yes	1060 (14.8)	539 (15.9)	521 (13.8)	0.006^1^
No	6048 (84.5)	2822 (83.2)	3226 (85.7)	
Don’t know	47 (0.7)	29 (0.9)	18 (0.5)	
**Do you have someone who can help you when you are ill? (%)**				
Yes, no problem	4468 (67.7)	2317 (71.1)	2151 (64.5)	< 0.001^2^
Yes, probably	1766 (26.8)	836 (25.7)	930 (27.8)	
No, probably not	244 (3.7)	79 (2.4)	165 (4.9)	
No, I do not	121 (1.8)	27 (0.8)	94 (2.8)	
**Number of individuals you know well enough that you can talk to them about everything (%)**				
None	149 (2.3)	51 (1.6)	98 (2.9)	< 0.001^2^
1–3	2369 (35.7)	1034 (31.6)	1335 (39.9)	
4–6	2134 (32.1)	1090 (33.3)	1044 (31.2)	
7–9	805 (12.2)	449 (13.7)	356 (10.6)	
10–15	707 (10.7)	386 (11.8)	321 (9.6)	
16–30	282 (4.3)	168 (5.1)	114 (3.4)	
> 30	176 (2.7)	95 (2.9)	81 (2.4)	
**Religious beliefs (%)**				
Yes	2868 (49.0)	1245 (44.0)	1623 (53.7)	< 0.001^1^
No	2984 (51.0)	1584 (56.0)	1400 (46.3)	
**Feeling lonely (%)**				
Yes, often	449 (6.4)	118 (3.5)	331 (9.1)	< 0.001^2^
Yes, sometimes	1863 (26.6)	740 (22.0)	1123 (30.9)	
No, seldom	1957 (28.0)	1071 (31.8)	886 (24.4)	
No, never	2729 (39.0)	1438 (42.7)	1291 (35.6)	
**Suicidal thoughts (%)**				
No suicidal thoughts	6310 (87.5)	3189 (93.5)	3121 (82.1)	< 0.001^1^
Life weariness	814 (11.3)	197 (5.8)	617 (16.2)	
Suicidal thoughts	89 (1.2)	23 (0.7)	66 (1.7)	

Chi-squared test was performed for nominal data^(1)^, the Mann-Whitney U-test for ordinal data^(2)^, and Student’s t-test^(3)^ for interval data. *SD* standard deviation, *Ys* years, *EUR* euros. The *p* value < 0.05 was used to test significance

Information about the individuals’ current thoughts on life weariness and suicide was collected in the questionnaire and measured through one of the nine items (item 9) derived from the Montgomery-Åsberg Depression Rating Scale (MADRS). The original response alternatives range from 0 to 6: 0 = Enjoys life or takes it as it comes; 2 = Weary of life. Fleeting suicidal thoughts; 4 = Probably better off dead. Suicidal thoughts are common. Suicide is considered a possible solution, but without specific plans or intention; 6 = Explicit plans for suicide. Active preparation for suicide [13]. More information about the MADRS scale and in what manner the item was used in the older population can be read elsewhere [12]. In the present study, suicidal expressions, including life weariness and suicidal thoughts, were classified into the following categories: (0) no life weariness or suicidal thoughts, [1, 2] life weariness, and [3–6] suicidal thoughts. The outcome variable of survival, i.e., date of death, was collected from the Swedish National Death Register.

### Data analysis

Descriptive statistics were used for variables in demographic data, such as social support, depression, and life weariness and suicidal thoughts, and given as absolute values and percentages (%). Chi-squared tests, the Mann-Whitney U-test, and the Student’s t-test were used to test the differences in the proportions at a nominal, ordinal, and interval data level, respectively. Participants were divided into three age groups (60–69, 70–79, and 80+ years) and age was combined with sex in an interaction variable. Survival time was calculated from the date of the baseline survey to the date of death during the 16-year follow-up, with time intervals presented in days: 0, 2000, 4000, and 6000 days, corresponding to 5.5, 11 and 16.4 years. To estimate the impact of the interaction of age and sex on survival, a Kaplan-Meier survival analysis was performed (for age group (60–69, 70–79, or 80+) and sex in combination) in the whole sample, and in those with and without life weariness and suicidal thoughts separately.

The differences between groups regarding mortality, sex-age interaction, and presence or absence of life weariness and suicidal thoughts, were tested and assessed using the log-rank test. To explore life weariness and suicidal thoughts together with the independent risk factors of mortality, the variables that showed a significant association in univariate analysis (sex-age interaction, marital status, education, housing, geographic area, feeling lonely, support from others, social contacts, and suicidal thoughts) were entered simultaneously into a multivariate Cox proportional hazard model (enter model). The confounders, country of birth, depression, and financial resources were included in the model as well. To verify the proportional hazard assumption, the cumulative hazard function was inspected visually. Models including adjusted variables were also created for individuals with and without life weariness and suicidal thoughts separately. Before entering the variables sex-age interaction, marital status, geographic area, country of birth, and social contacts, dummy variables were used to distinguish between different groups. The likelihood ratio significance test was used to assess goodness-of-fit for the models created. Cox proportional hazard models were used to calculate the multi-adjusted hazard ratio (HR) with 95% confidence interval (CI) of mortality in a long-term perspective, with a particular focus on life weariness and suicidal thoughts at the baseline examination. The population attributable risk (PAR) of life weariness and suicidal thoughts was calculated with HR [20]. SPSS 25.0 (IBM SPSS Statistics for Windows, Version 25.0. Armonk, NY: IBM Corp., USA) was used in all analyses, and the level of significance was set to *p* <  0.05.

## Results

At baseline, the study sample included 7213 participants (59.5% females), with a mean age of 73.11 years (SD 10.72). A total of 3804 (52.7%) individuals died during the observation period. The 80+ females had the highest proportion of deaths (38.1%), twice as high as the 80+ males (18.5%). Compared with the individuals in the deceased group, the survivors differed significantly in all variables included, except in the variable of financial difficulties during the last year.

In the total sample, 12.5% of the individuals reported life weariness and suicidal thoughts at baseline. Fewer individuals in the survival group reported life weariness and suicidal thoughts compared with those in the deceased group (6.5% vs. 17.9%). At baseline, more females and males in the oldest age group (80+) had life weariness and suicidal thoughts (23.0 and 17.9%, respectively) than females and males in the youngest age group (60–69) (8.1 and 4.9%, respectively) (*P* <  0.001). Demographic characteristics of the survivors and the deceased are shown in Table 1.

**Fig. 1:**
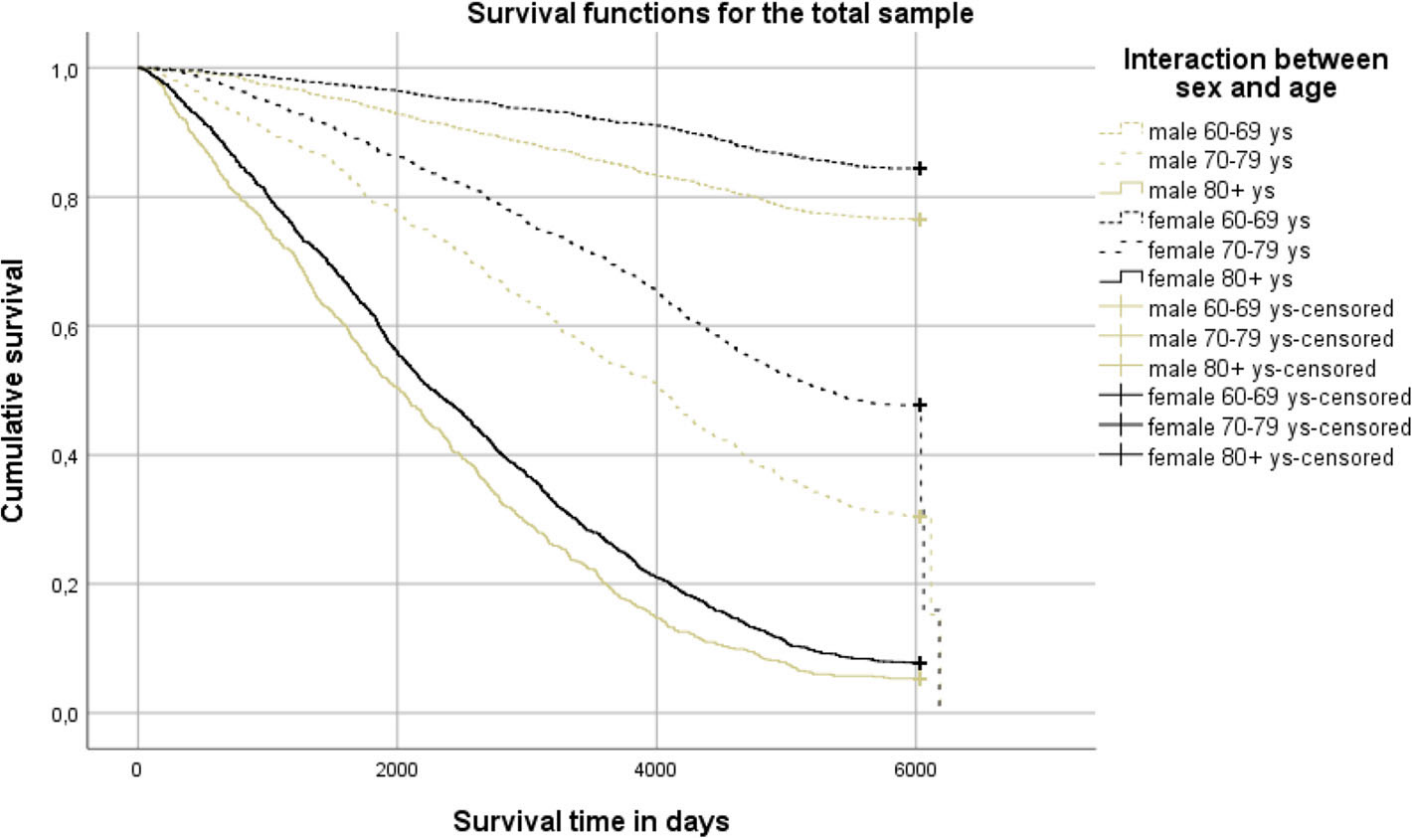
Survival curves and life 18 table, for the total sample (log rank test, *p* value < 0.001)

**Fig. 2:**
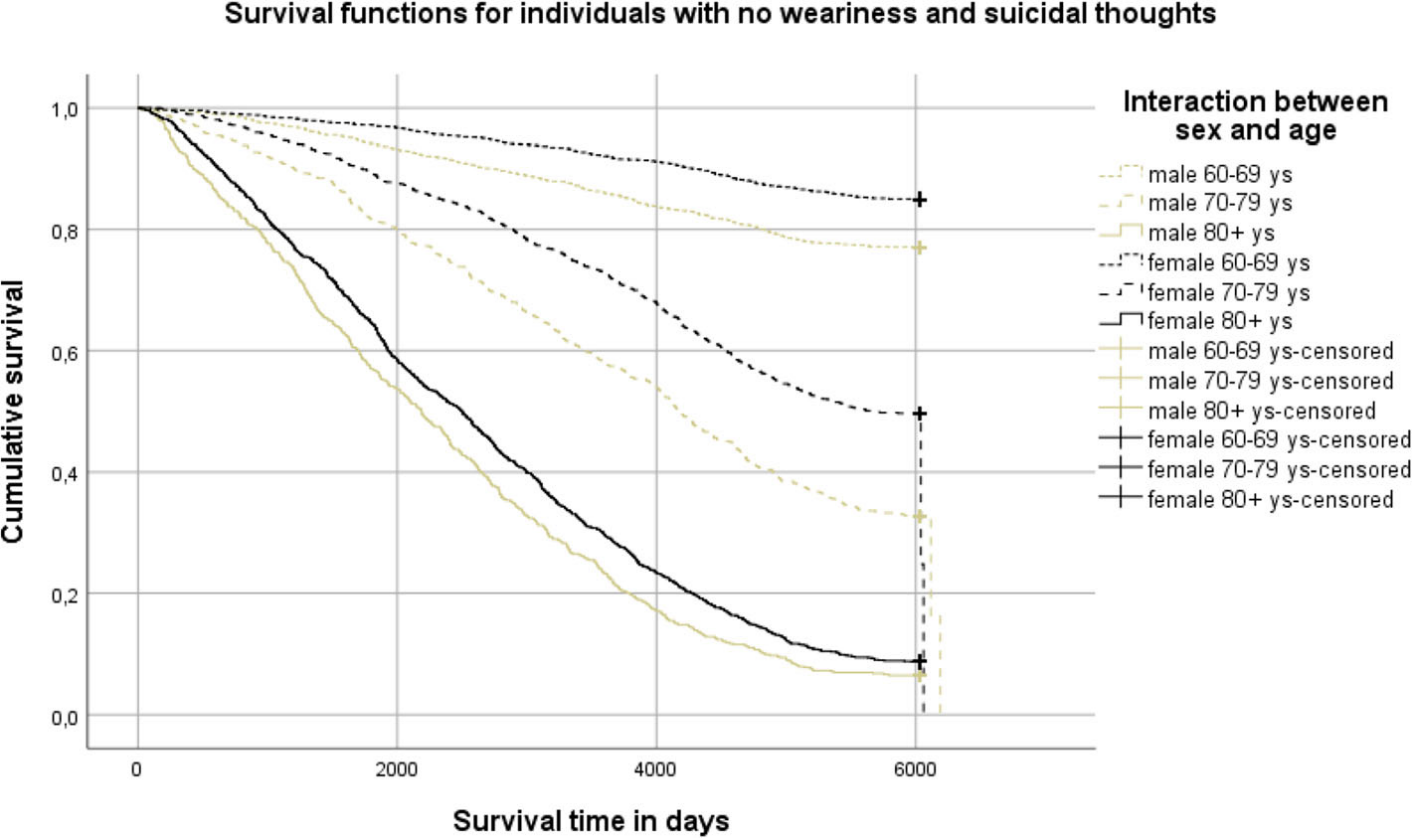
Survival curves and life table, for those with no life weariness and suicidal thoughts (log rank test, p value < 0.001)

**Fig. 3:**
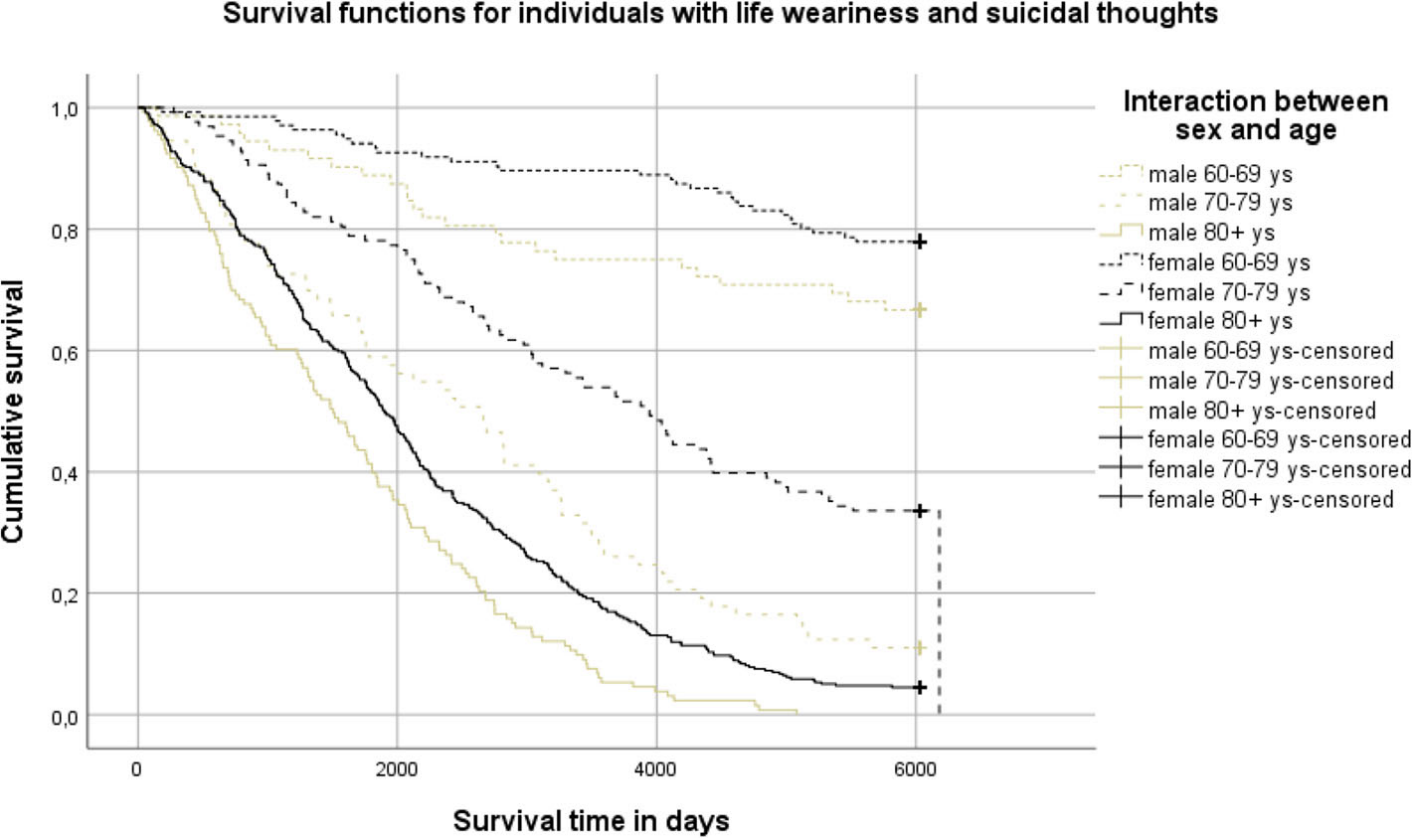
Survival curves and life table, for those with life weariness/suicidal thoughts (log rank test, p value < 0.001)

Individuals with life weariness and suicidal thoughts had half the survival rate (24.5% vs. 50.6%) of those without such thoughts, with a survival time of 8.4 years (SD 5.7) versus 12.0 years (SD 5.4). The six groups of sex-and age interactions differed significantly in survival rate in the total sample and in individuals with and without life weariness and suicidal thoughts, respectively (log-rank test *P* <  0.001). In the group of individuals with no life weariness and suicidal thoughts, males in the age group 80+ showed the lowest survival rate (6.4%) and survival time (mean 6.6 years, SD 4.5) in comparison with any of the combinations female 80+ years (8.7%, mean 7.4 years, SD 4.7), male 70–79 years (32.6%, mean 10.9 years, SD 5.2), female 70–79 years (49.6%, mean 12.6 years, SD 4.8), male 60–69 years (77.0%, mean 14.6 years, SD 4.0), or female 60–69 years (85.0%, mean 15.4 years, SD 3.1). In the group of individuals with life weariness and suicidal thoughts, males 80+ years also showed the lowest survival rate and survival time (0%, mean 4.5 years SD 3.2) compared with females 80+ years (4.4%, mean 6.0 years, SD 4.2), males 70–79 years (11.0%, mean 7.3 years, SD 5.0), females 70–79 years (33.6%, mean 10.4 years, SD 5.5), males 60–69 years (66.7%, mean 13.4 years, SD 5.2), or females 60–69 years (77.6%, mean 14.9 years, SD 3.7). Separate survival curves for sex-age interactions in individuals in the total sample and in those with or without life weariness and suicidal thoughts at baseline, respectively, and numbers of people grouped by risk exposures, are presented in Figs. 1, 2 and 3.

**Fig. 4:**
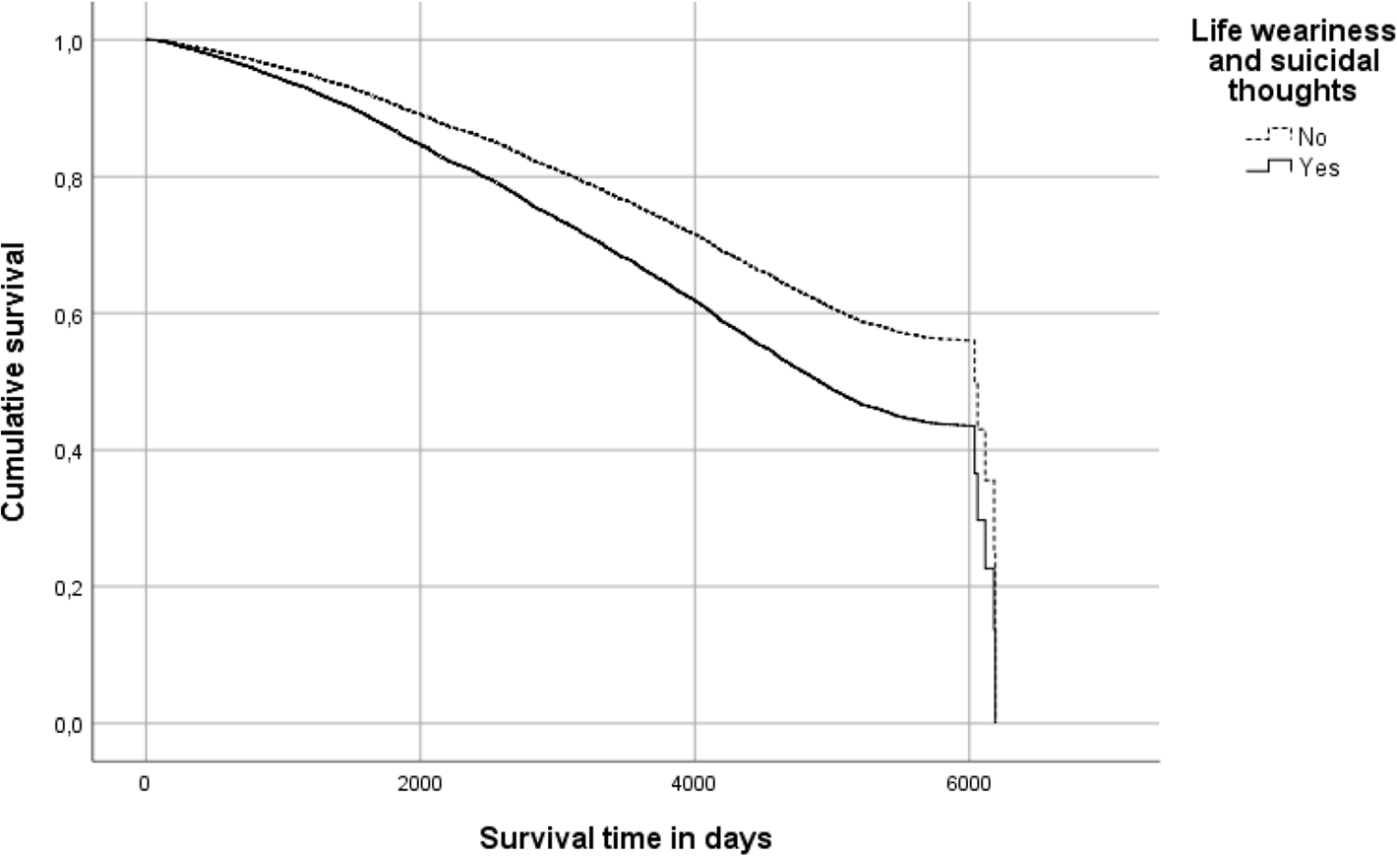
Survival curves for life weariness and suicidal thoughts, adjusted for control factors. Note: The time intervals are presented in days: 0, 2000, 4000, and 6000 days, corresponding to 5.5, 11 and 16.4 years

The results from the Cox proportional hazard models (univariate and multivariate) showed significant associations between life weariness and suicidal thoughts and survival time. In the univariate Cox proportional hazard model, the HR of mortality was 1.42 (95% CI 1.31–1.55, *P* <  0.001) for those who reported life weariness and suicidal thoughts and the HR of mortality remained when adjusting for the control factors included. The multi-adjusted HR of mortality for those who reported life weariness and suicidal thoughts was 1.44, 95% CI 1.30–1.59 (*P* <  0.001) (Table 2). PAR was 11.1%, where those with life weariness and suicidal thoughts had a lower survival rate and shorter survival time (log-rank test, P <  0.001). Males and females 80+ years (ref. female 60–69 years) had the highest multi-adjusted HRs of mortality: 17.79 and 11.21, respectively (95% CIs 15.13–20.90 and 9.60–13.08, respectively). The HRs of the sex-age interactions (male and female 80+ years) in relation to mortality remained high in the stratified hazard models for life weariness and suicidal thoughts, especially in individuals with life weariness and suicidal thoughts at baseline (HR males 22.39, CI 13.85–36.18; HR females 12.49, CI 7.93–19.66) (Table 3). Figure 4 shows the survival curves for individuals with and without life weariness and suicidal thoughts, adjusted for the control factors included (demographic factors, social network factors, and depression).

**Table 2:** Hazard ratios for life weariness/suicidal thoughts, adjusted for control factors included

	HR	CI	*p* value
Life weariness and suicidal thoughts			
No	Ref	-	**-**
Yes	1.44	1.30–1.59	**< 0.001**
Sex-age interaction			
Female 60–69 ys	Ref	-	**-**
Female 70–79 ys	3.86	3.29–4.54	**< 0.001**
Female 80+ ys	11.21	9.60–13.08	**< 0.001**
Male 60–69 ys	1.77	1.49–2.10	**< 0.001**
Male 70–79 ys	7.42	6.30–8.74	**< 0.001**
Male 80+ ys	17.79	15.13–20.90	**< 0.001**
Education			
Primary school	Ref	-	
Secondary school or higher education	1.13	1.05–1.23	**0.002**
Marital status			
Married	Ref	-	**-**
Widow/-er	1.34	1.21–1.48	**< 0.001**
Unmarried	1.34	1.18–1.54	**< 0.001**
Divorced	1.22	1.07–1.40	**0.003**
Living apart	0.97	0.74–1.28	0.828
Housing			
Community dwelling	Ref	-	**-**
Residential care facility or other	1.68	1.41–2.00	**< 0.001**
Geographic area			
Urban	Ref	-	**-**
Semi-urban	1.07	0.99–1.16	0.100
Rural	1.15	0.87–1.52	0.319
Financial difficulties in the past year			
No	Ref	-	-
Yes	1.06	0.99–1.25	0.502
Able to access EUR 1,400 within a week			
Yes	Ref	-	-
No	1.23	1.11–1.36	**< 0.001**
Feeling lonely			
No	Ref	-	-
Yes	1.09	1.00–1.18	0.051
Country of birth			
Sweden	Ref	-	**-**
Other Nordic country	1.34	1.10–1.63	**0.003**
Other European country	0.82	0.68–0.99	**0.043**
Outside Europe	1.02	0.78–1.35	0.882
Depression			
No	Ref	-	-
Yes	1.01	0.91–1.12	0.893
Do you have someone who can help you when you are ill?			
Yes	Ref	-	-
No	1.12	0.98–1.29	0.100
Number of individuals you know well enough that you can talk to them about everything			
≥ 10	Ref	-	-
4–9	1.10	0.99–1.22	0.087
0–3	1.12	1.01–1.25	**0.040**

In the univariate Cox regression model, the HR for mortality was 1.42 (95% CI 1.31-1.55) in those reported life weariness and suicidal thoughts (*p* < 0.001). Log likelihood *p* value < 0.001. Cox analyses with enter model. Significant factors in bold. *HR* hazard ratio, *CI* confidence interval, *EUR* euros

**Table 3:** Hazard ratios for control factors: without and with life weariness/suicidal thoughts

	Without life weariness and suicidal thoughts			With life weariness and suicidal thoughts		
	HR	CI	*p* value	HR	CI	*p* value
**Sex-age interaction**						
Female 60–69 ys	Ref	**-**	**-**	Ref	**-**	-
Female 70–79 ys	**3.83**	**3.22–4.55**	**< 0.001**	**4.65**	**2.87–7.54**	**< 0.001**
Female 80+ ys	**11.27**	**9.54–13.31**	**< 0.001**	**12.49**	**7.93–19.66**	**< 0.001**
Male 60–69 ys	**1.77**	**1.47–2.12**	**< 0.001**	**1.87**	**1.04–3.36**	**< 0.001**
Male 70–79 ys	**7.25**	**6.09–8.63**	**< 0.001**	**9.77**	**5.93–16.08**	**< 0.001**
Male 80+ ys	**17.20**	**14.47–20.45**	**< 0.001**	**22.39**	**13.85–36.18**	**< 0.001**
**Education**						
Primary school	Ref	-	-	Ref	-	-
Secondary school or higher education	**1.12**	**1.02–1.22**	**0.013**	**1.22**	**1.00–1.48**	**0.047**
**Marital status**						
Married	Ref	-	-	Ref	-	-
Widow/-er	**1.38**	**1.24–1.54**	< **0.001**	1.10	0.86–1.39	0.452
Unmarried	**1.41**	**1.22–1.63**	**< 0.001**	0.91	0.64-1.31	0.613
Divorced	**1.21**	**1.04–1.41**	**0.013**	1.34	0.80–1.62	0.485
Living apart	0.96	0.72–1.28	0.783	1.03	0.37–2.83	0.960
**Housing**						
Community dwelling	Ref	-	-	Ref	-	-
Residential care facility or other	**1.53**	**1.25–1.87**	**< 0.001**	**2.23**	**1.57–3.18**	**< 0.001**
**Geographic area**						
Urban	Ref	-	-	Ref	-	-
Semi-urban	**1.13**	**1.04–1.24**	**0.005**	**0.78**	**0.64–0.95**	**0.015**
Rural	1.17	0.88–1.56	0.284	1.45	0.45–4.65	0.532
**Financial difficulties in the past year**						
No	Ref	-	-	Ref	-	-
Yes	1.04	0.86–1.24	0.714	1.30	0.88–1.92	0.189
**Able to access EUR 1,400 within a week**						
Yes	Ref	-	-	Ref	-	-
No	**1.22**	**1.08–1.38**	**0.001**	1.22	0.97–1.53	0.089
**Feeling lonely**						
No	Ref	-	-	Ref	-	-
Yes	**1.12**	**1.03–1.23**	**0.012**	0.97	0.79–1.18	0.738
**Country of birth**						
Sweden	Ref	-	-	Ref	-	-
Other Nordic country	**1.35**	**1.09–1.67**	**0.007**	1.24	0.75–2.05	0.403
Other European country	0.83	0.67–1.02	0.079	0.90	0.57–1.42	0.644
Outside Europe	0.99	0.73–1.33	0.925	**4.13**	**1.51–11.34**	**0.006**
**Depression**						
No	Ref	-	-	Ref	-	-
Yes	0.92	0.82–1.04	0.192	**1.26**	**1.02–1.57**	**0.034**
**Do you have someone who can help you when you are ill?**						
Yes	Ref	-	-	Ref	-	-
No	1.15	0.98–1.35	0.079	1.04	0.78–1.37	0.799
**Number of individuals you know well enough that you can talk to them about everything**						
≥ 10	Ref	-	-	Ref	-	-
4–9	1.11	0.99–1.24	0.67	0.89	0.63–1.26	0.738
0–3	**1.12**	**1.00–1.26**	**0.50**	0.98	0.70–1.39	0.508

Log likelihood *p* value < 0.001. Cox regression analyses with enter model. Significant factors in bold. *HR* hazard ratio, *CI* confidence interval, *EUR* euros

## Discussion

This study, based on a large, geographically diverse, national sample of an older population, investigated the effect of life weariness and suicidal thoughts on mortality when controlling for expected age-related factors. A total of 12.5% of participants reported life weariness and suicidal thoughts at baseline. About half of the individuals had died at the end of the study and interaction of sex and higher age increased the risk of death. Our analysis has added value concerning our understanding of the effect of life weariness and suicidal thoughts on mortality in older adults. For example, being an individual with life weariness and suicidal thoughts was independently associated with a 1.44 times higher risk (hazard ratio) of death within 16 years, in comparison with those without such thoughts. Individuals with life weariness and suicidal thoughts had a shorter mean survival time (3.6 years) and their survival rate was half that of other individuals (24.5% vs. 50.6%).

Older adults have previously been found to be particularly vulnerable to health problems due to reduced internal and external resources [21], factors included in the present study. Reduced resources increase the complexity of meeting, identifying, treating, and studying older individuals regarding suicidality. The high mortality rate found in this study underscores the importance of detecting life weariness and suicidal thoughts in older people. Furthermore, the findings indicate that subtle and early signs of suicidality, such as life weariness and suicidal thoughts, need to be assessed more structurally and thoroughly in old age, especially within community and primary care settings. By detecting life weariness and suicidal thoughts through screening, identifying causes, and developing solutions, we could improve preventive strategies and treatment, and thereby reduce the risk of mortality.

With the expected increase of life weariness and suicidal thoughts in the population due to increased demographic aging, it is vital to understand early signs and risk factors for mortality. The present study contributes to the understanding of the possible long-term impact of demographic and social factors on mortality in older adults. Our study is supported by earlier evidence on the elevated risk of death in older adults with suicidal thoughts [14, 15]. A recent Korean literature review pointed out that multi-level factors, including individual, family, and social aspects, can interact over a lifetime to protect against suicidal thoughts in older adults [22]. This highlights the importance of including several factors over time in research. However, there are still few robust studies from other countries and under other conditions, making it difficult to validate results.

Regarding the demographic factors, the present study confirmed several aspects of the sex and age interaction with suicidality established by previous research, most of which was cross-sectional. In this study, more females (8%) than males (3.7%) had life weariness and suicidal thoughts at baseline (*P* <  0.001). The highest proportion of life weariness and suicidal thoughts was found in the group of females 80+ years (23.0%), followed by males in the same age group (17.9%). The results regarding sex are supported by Barnow et al. [23] and Barnow and Linden [1], but not by Almeida et al. [24] and Ko et al. [25], who found a higher proportion among men. Interestingly, when investigating the predictive value of the sex-age interaction for mortality, we found that males, not females, 80+ years with life weariness and suicidal thoughts, had the highest multi-adjusted hazard ratio of mortality (22.4, CI 13.85–36.18). In previous literature, there is no clear consensus on if the sex of older adults has a potential relationship with suicidality. For instance, it has been found that older males are at higher risk of suicide than females [3, 24, 26–29], while females report more suicidal thoughts compared to males [1, 23, 30, 31] but also that there are no sex differences as regards suicidal thoughts [12]. Here, the varying results may be due to the use of different spectrums of the concept, the contexts, and the measurements of suicidality in the studies of older populations, as well as differences in the ages of the populations included. This complexity justifies the assumption of moderation through multiple factors, and confirms the relevance of including socio-demographic data to verify factors contributing to mortality.

Another important finding of the present study was that life weariness and suicidal thoughts increased with higher age. This finding is in agreement with prior research showing that suicidal thoughts increase in older adults [10]. In addition, the highest difference in survival rate was found in males 70–79 years, where those without life weariness and suicidal thoughts had 3.6 years longer mean survival time (10.9 years vs. 7.3 years, *P* value < 0.001) than those with life weariness and suicidal thoughts. We did not specifically investigate the risk of death by completed suicide, which is higher in older males, but our study indicates that being a male, especially of high age, with suicidal thoughts, increased the risk of death. It should be noted that our study, like previous research, asked only about legal sex (i.e., male or female) and we lack an understanding of how gender identification potentially affects the results presented here.

Several other independent predictors, aside from life weariness, suicidal thoughts and sex-age interactions, had varying yet significant importance for mortality in the models separated for individuals with and without life weariness and suicidal thoughts (cf. Table 3). Living in a residential care facility, being born in a country outside Europe, not being married, having insufficient financial resources, having low education, and having an insufficient social network should also be considered in the discussion of mortality and life weariness and suicidal thoughts in older adults. Although not investigated here, it may not be living in a residential care facility that increases the risk of death, but declined physical and mental health and functional ability and a lack of social relationships. The latter are more common in people living in residential care facilities than in those living in ordinary homes. Holt-Lunstad et al. [32] and Yasuda et al. [33] also saw the importance of social network contacts for mortality, but found that contacts decreased with age. The number of people that a person feels they can trust has also, in previous literature, been associated with mortality in old age [34, 35]. In Sweden, about half of those in the older population (80+) are living alone [36]. Furthermore, in the age group 80+, more women than men are living alone and about 80% of them are widows [37], who often have restricted financial resources and low education, i.e., factors connected to mortality in old age [38]. Such circumstances need to be considered when striving to understand differences in survival in older adults, as well as the varying importance of these factors in the groups with and without life weariness and suicidal thoughts.

In the literature, there are conflicting results regarding whether the effect of suicidal thoughts on mortality in older adults remains after controlling for factors such as depression [15]. The findings in the present study suggest that life weariness and suicidal thoughts have an effect on mortality in old age, after controlling for depression. This is in line with two other longitudinal studies which found that suicidal thoughts had a significant association with mortality after controlling for various factors, including depression [14, 15]. This finding has important implications for targeted preventive actions in old age mental health to decrease mortality. A systematic literature review focusing on preventive interventions for suicidal thoughts in older adults found that most studies have targeted depression as a risk factor, for example through primary care depression screening and management [39]. Such interventions are important, but may not be sufficient to prevent suicidal thoughts and mortality in the long term. Furthermore, previous research has found depression to be a strong influential factor for suicidality in old age (see for example [24, 28, 40]). Death by suicide was not investigated in the present study, yet our results showed that depression had an effect on mortality in the case of individuals reporting life weariness and suicidal thoughts. The effects of life weariness, suicidal thoughts, and depression on mortality are undoubtedly complex, with difficulty determining the potential reverse effects of mortality on depression and suicidal thoughts. Still, considering the elevated risk of death in individuals with life weariness and suicidal thoughts and without depression in the present study, more research into preventive interventions is needed.

The strengths of this study included the prospective design, with the possibility to investigate the long-term effects on mortality in a large sample, and the nationally representative sample. The study also included several demographic and social confounders which are relevant in advanced age and can be expected to influence the association between suicidal thoughts and mortality. Furthermore, the sample included individuals from both urban and rural areas, and from both ordinary homes and residential care facilities, meaning that the result can be discussed and generalised to a larger old-age population. Limitations might include that we had no information about the individuals who participated at baseline but were lost at the time of the follow-up. Also, the use of self-reported life weariness and suicidal thoughts might have introduced a bias by underreporting, due to normalisation and non-recognition [3] of the symptoms in late life. This may have led to a misclassification of the risk group. Moreover, although the item taken from the MADRS scale comprises response options ranging from life weariness to explicit plans and active preparation for suicide, the original item used is described only as suicidal thoughts by the authors [13], which could be misleading in the conceptualisation of the concept (cf. 12). Furthermore, the ratings in MADRS were based on the individuals’ current thoughts of life weariness and suicidal thoughts, meaning that the timepoint might have been uncertain.

Among the individuals randomly invited to participate in SNAC, the most common reason not to participate was poor health. It might be a limitation that only depression was included in the analysis as a well-known predictor of suicidal thoughts. Furthermore, the information about depression was self-reported and not verified through diagnosis. There is reason to believe that diseases other than depression may have a relationship with life weariness and suicidal thoughts and would be relevant to include, especially considering that the variable residential home facility was an independent risk factor for mortality. However, the potential importance of health factors was outside the scope of the present study. Approximately 3% (*n* = 203) of the included individuals declined to share information about life weariness and suicidal thoughts, meaning that the rate of individuals with life weariness and suicidal thoughts could be underrepresented. However, the proportion found (12.5%) replicated previous findings of suicidal expressions, where about 13% experienced life weariness [41]. This study did not aim to investigate specific causes of death and further research are needed to understand the causal mechanism between suicidality and survival.

## Conclusions

This study is, to our knowledge, the first to investigate the relationship between life weariness and suicidal thoughts and survival in older adults, using a large and nationally representative sample over a long time period. The findings underlined life weariness and suicidal thoughts as risk factors for long-term mortality. Individuals with life weariness and suicidal thoughts had half the survival rate and a shorter survival time compared with those without such thoughts. This study also showed that the interaction between sex and age predicted long-term mortality among older adults, especially in individuals with life weariness and suicidal thoughts. The findings suggest that screening, identifying, and intercepting early signs of suicidality in older adults with life weariness and suicidal thoughts could improve the survival rate in the older population.

## Data Availability

The public access to the database SNAC is closed. We have received administrative permission to access and use data in SNAC from the Primary Investigators (PI) of the three cites included (SNAC Blekinge, GÅS and Kungsholmen). All data generated or analysed during this study are included in this published article.

## Abbreviations

CI: confidence interval
GÅS: Good Aging in Skåne
HR: hazard ratio
LR: likelihood ratio
MADRS: Montgomery-Åsberg Depression Rating Scale
PAR: the population attributable risk
SD: standard deviation
SNAC: Swedish National Study on Aging Care
